# Mismatched ambition, execution and outcomes: implementing maternal death surveillance and response system in Mtwara region, Tanzania

**DOI:** 10.1136/bmjgh-2021-005040

**Published:** 2021-05-20

**Authors:** Ali Said, Nathanael Sirili, Siriel Massawe, Andrea B Pembe, Claudia Hanson, Mats Malqvist

**Affiliations:** 1 Department of Women and Children’s Health, Uppsala University, Uppsala, Sweden; 2 Department of Obstetrics and Gynecology, Muhimbili University of Health and Allied Sciences, Dar es Salaam, Tanzania, United Republic of; 3 Department of Development Studies, Muhimbili University of Health and Allied Sciences, Dar es Salaam, Tanzania, United Republic of; 4 Department of Global Public Health, Karolinska Institute, Stockholm, Sweden; 5 Department of Disease Control, London School of Hygiene and Tropical Medicine, London, UK

**Keywords:** health systems, health systems evaluation, maternal health, obstetrics, health services research

## Abstract

**Background:**

Since 2015, Tanzania has been implementing the Maternal Death Surveillance and Response (MDSR) system. The system employs interactions of health providers and managers to identify, notify and review maternal deaths and recommend strategies for preventing further deaths. We aimed to analyse perceptions and experiences of health providers and managers in implementing the MDSR system.

**Methods:**

An exploratory qualitative study was carried out with 30 purposively selected health providers and 30 health managers in four councils from the Mtwara region between June and July 2020. Key informant interviews and focus group discussions were used to collect data. Inductive thematic analysis was used to analyse data.

**Results:**

Two main themes emerged from this study: ‘Accomplishing by ambitions’ and ‘A flawed system*’*. The themes suggest that health providers and managers have a strong desire to make the MDSR system work by making deliberate efforts to implement it. They reported working hard to timely notify, review death and implement action plans from meetings. Health providers and managers reported that MDSR has produced changes in care provision such as behavioural changes towards maternal care, increased accountability and policy changes. The system was however flawed by lack of training, organisational problems, poor coordination with other reporting and quality improvements systems, assigning blame and lack of motivation.

**Conclusion:**

The implementation of the MDSR system in Tanzania faces systemic, contextual and individual challenges. However, our results indicate that health providers and managers are willing and committed to improve service delivery to avoid maternal deaths. Empowering health providers and managers by training and addressing the flaws will improve the system and quality of care.

**Key questions**

**What is already known?**

- Tanzania has one of the highest maternal mortality ratio in the world.
- Tanzania introduced a system of tracking and reviewing circumstances of maternal deaths inorder to improve quality of care to prevent future deaths.

**What are the new findings?**

- Implementers are committed and motivated to reduce maternal deaths by making sure the system works as intended
- Implementation of the system faces challenges of lack of training, blame culture, poor supervision and poor coordination with other systems.
- It has had notable impact on quality of care, accountability and policy changes.

**What do the new findings imply?**

- The system has significant support from implementers.
- Addressing the challenges facing the system will improve quality of care and reduce maternal deaths.

## Background

Worldwide, maternal mortality is still at an unacceptably high level, with about 295 000 maternal deaths counted in 2017.^1–3^ The number of maternal deaths differs significantly between different regions, with most occurring in low-income countries. In Tanzania, maternal mortality is estimated at 524/100 000 live births in 2017 according to the WHO^3^ which is higher than the 2012 census estimation of 432 per 100 000 live births.^4^ In 2015, the maternal death surveillance and response (MDSR) system was introduced in Tanzania, following a 20-year period of implementing maternal deaths audits. The purpose was to improve quality of care and reduce maternal deaths in line with WHO recommendations. The MDSR system theoretically links the health information system and quality improvement processes at community, facility and national levels through a continuous process. It was initiated through an introduction of guidelines and a training-of-trainers scheme in each region, with regional trainers being instructed to cascade the training to district and facility health providers and managers.^5^ Through the MDSR system, maternal deaths should be identified and notified within 24 hours of their occurrence. Then MDSR committees at the facilities where the deaths occurred should meet and confidentially discuss and reflect on the events leading to deaths. Discussions and evaluations should be conducted in an open and honest manner, without assigning blame or pointing at individual mistakes.^5^ Quality improvement recommendations ought to address the identified gaps in care provision, in order to positively affect maternal deaths prevention efforts.

**Figure 1:**
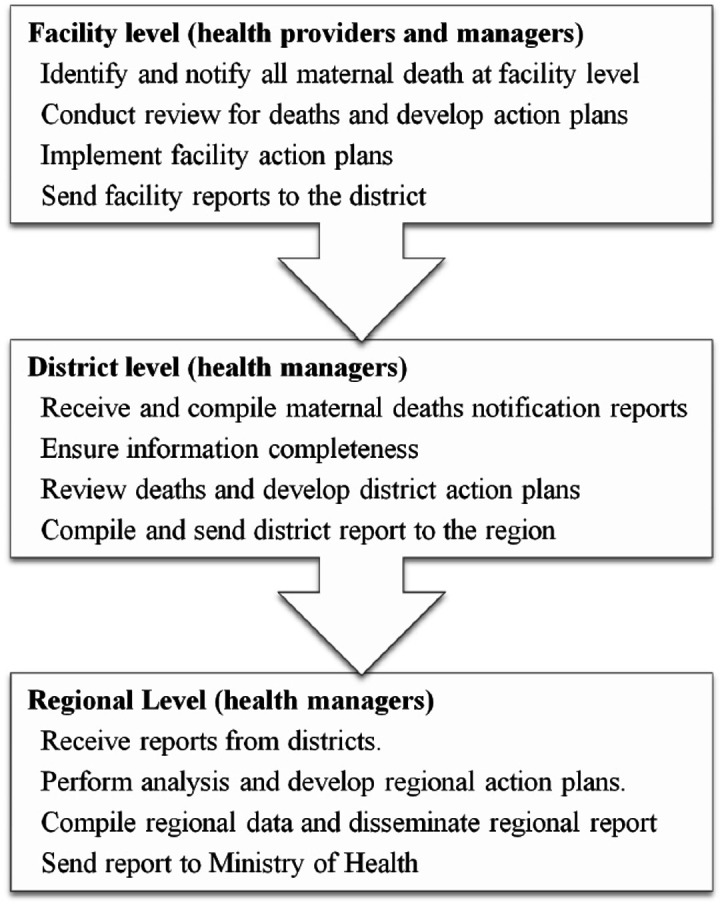
Shows the responsibilities of health providers and managers in the MDSR system. The mainly involve notification, review of deaths and sending reports and receiving feedback from higher level of the health system. MDSR, maternal death surveillance and response.

To accomplish its objectives, the MDSR system relies on complex interactions between health managers, health providers, government leaders, community members and other stakeholders, each with a specific role. For example, health managers mostly supervise activities and follow-up notification of deaths and implementation of action plans. The health providers implement most activities such as notification and review of deaths and sending periodic reports (figure 1). Implementers have more than one task and sometimes they overlap. This complex interaction can affect the system in both a positive and a negative way. For example, integration with other systems, such as notification systems and quality improvement teams, is one of the cornerstones to make MDSR successful.^6^

However, reports on MDSR from Sudan and Bangladesh point to implementation constraints such as poor integration and separation from health information system, missing information in medical records and inadequate health providers’ skills^7 8^ and in Northern Tanzania, quality improvement teams in health facilities were found to work separately from MDSR teams, resulting in fragmented implementation of recommendations.^9^ Another study that analysed data from MDSR systems in multiple countries found that an established system of maternal death notification and facility review was present at most places. There were, however, a lot of missing links between the review information and response to the maternal deaths due to lack of governance and accountability among reviewers and policy-makers.^10^ This lack of integration might have caused implementation of actions that did not target gaps identified in the MDSR system.

Other challenges to the successful implementation of the MDSR system have been described in studies from Eastern and Southern Africa. Lack of knowledge, legal framework and accountability were reported to hinder MDSR activities.^11^ Furthermore, inadequate resources to perform and support MDSR, inadequate community involvement and lack of follow-up on recommendations were also reported to affect the implementation in these countries.^11 12^ In Malawi, health providers reported feeling discouraged by the MDSR process because of experiences of being yelled at and blamed, and they expressed that the system’s focus on mistakes only led to finger pointing during the review meetings.^13^ Most health providers may shy away from the process when they constantly meet an environment of being blamed. There is however little literature on how implementers in Tanzania (health providers and managers) have experienced the process and what challenges have affected the system since its initiation. Previous studies were done before the introduction of MDSR in Tanzania. Some of these focused on document reviews but not on health providers’ and managers’ experiences. Challenges on MDSR implementation have mostly been reported in other settings therefore there is scarce data of Tanzanian context. Two studies done recently on Tanzanian MDSR system reported strengths and challenges in reporting causes of death, delays in care provision and comprehensiveness of documents.^14 15^ We also aimed to explore reasons for the shortcomings identified by these studies from providers and managers perspectives. Therefore, study aims to analyse health providers’ and managers’ perceptions and experiences of the implementation of the MDSR system in Tanzania.

## Methods

### Study design

An exploratory qualitative study design with focus group discussions (FGDs) and key informant interviews (KIIs) at regional, district and facility levels was applied. FGDs were conducted among health providers in the wards who were members of MDSR committees while KIIs focused on health managers experiences. These methods were used to makes sure that rich data were collected from both health providers and managers. There are many MDSR committee members with different experiences on MDSR depending on their cadre, experience in maternal care and participation in the system’s activities. FGDs were used among these health providers in order to explore their perceptions and experiences with MDSR, however, little they had. Key informants interviews were used among health managers in order to explore their experiences more deeply since they have been implementing the system for a longer time than most health providers. Another reason was that logistically it would have been difficult to organise FGDs with managers.

### Study context

The study was conducted in four districts in the Mtwara region of Southern Tanzania, where the two districts with the highest number of maternal deaths in the year 2018 (Masasi District Council and Mtwara Town Council) and the two districts with the lowest number (Nanyamba and Tandahimba District Councils) were selected for the study. All facilities, public and private (dispensaries, health centres, district and regional hospitals) that conduct delivery services are required to implement the MDSR system in Tanzania. The dispensary is at the lowest level, which serves local residents in villages/streets and refers patients to health centres which serve local wards. The health centres refer patients to district hospitals, which in turn refer patients to regional hospitals. All levels of facilities are capable of providing antenatal care and delivery services. Caesarean sections are performed in all hospitals but only a few health centres. Mtwara has a total population of about 1.2 million people according to the 2012 census.^4^ The region has a well-established MDSR system in all its’ districts since 2015. These facilities have multidisciplinary MDSR committees consisting of obstetricians, medical doctors, clinicians, nurses, midwives, laboratory personnel, pharmacy staff, drivers and other supporting staff, which reviews all maternal deaths. The size and structure of the committees depends on the level of the facility, and the number and availability of staff. The medical officer in-charge is the chairperson of the hospital MDSR committee. When a death occurs at the facility, notification is sent to the district and regional Reproductive Child Health Coordinators. The facility MDSR committee reviews the death within 7 days and fills the maternal death reporting form which is sent to the district and regional health office together with a narrative summary and action plans put forward by the committee. The region and each district have quarterly MDSR meetings to discuss maternal deaths happening in the quarter and make regional and district recommendations. These are implemented and reports are sent to the Ministry of Health, Community Development, Gender, Elderly and Children (MoHCDGEC) (figure 1).

### Sampling of study participants

Key informants were selected from one Regional Health Management Team (RHMT), two District Executive Directors’ (DED) offices, four Council Health Management Teams (CHMT) and four health facilities. Participants were purposively sampled on the basis of their position in the health system, as well as their participation in MDSR activities for at least 6 months. Furthermore, snowball sampling was used to include more key informants from other facilities. In total, 30 key informants from the DEDs office, RHMT, CHMTs and health facilities were included (table 1).

**Table 1:** Demographic characteristics of the participants (N=60)

Demographic characteristics	Key informants (n=30)	FGD participants (n=30)
District		
Mtwara Municipal Council	12	8
Nanyamba District Council	6	8
Tandahimba District Council	6	8
Masasi District Council	6	6
Cadre of the respondent		
Clinician*	9	5
Nurse	5	10
Nurse midwife	11	5
Obstetrician	2	0
Pharmacist	0	3
Lab technician	0	4
Anaesthetist	0	1
Other	3	2
Sex		
Male	11	19
Females	19	11
Experience with MDSR (months)		
8–24	14	23
25–48	11	4
>48	5	3
Age groups		
21–30	3	11
31–40	14	14
41–50	6	3
51–57	7	2

Others (teacher, lawyer, health secretary and social welfare).

*Includes medical doctors and assistant medical officers.

FGD, focus group discussion; MDSR, maternal death surveillance and response.

A total of four FGDs with 6–8 participants were conducted; one from each selected health facility in the four districts. These included members of facility MDSR committees who were not health managers, such as clinicians and nurses from maternity/labour wards, pharmacy staff and laboratory staff. We specifically excluded health managers in the FGDs in order to avoid instances where health providers might not reveal some information due to fear of their managers. Participants were selected by information provided by those in charge at facilities or hospital matron. We worked closely with these managers who identified all members of the committee in the facility on the day of data collection. We used our inclusion criteria to select participants for FGDs. Participants were selected on basis of being an MDSR committee member, having participated in MDSR activities for at least 6 months and were excluded if they were health managers. In total, 30 health providers from MDSR committees participated in the FGDs (table 1).

Most of the participants were clinicians, nurses and nurse midwives. Half of them were females, most (28) were aged 31–40 years and most (37) had 6–24 months experience with MDSR activities (table 1).

### Data collection

Data collection in each district started with KIIs where health managers from management teams, facility leaders and government officials were interviewed. It was followed by FGDs with facility (hospital/health centre) MDSR committee members. Discussions focused on how the MDSR was initiated in the region/district/facility, the dissemination and role of guidelines, how participants were initiated and introduced to their roles, how notification of deaths and the review process are currently done, and how action plans are formulated and implemented. FGDs also included discussions of barriers and facilitators of the MDSR system, its initiation and current status. Furthermore, issues on community maternal deaths and reviews were explored. The interview guide included open ended questions with probes and focused on the MDSR implementation cycle. All interviews were conducted in Swahili and were audio recorded. KIIs lasted for approximately 45–60 min while FGDs were done for 60–120 min.

The data collection process was inspired by Lincoln and Guba following an emergent design where data collection was done concurrently with continuous data analysis.^16 17^ Preliminary results were shared with the research team in the field after each day’s interviews, and all interviewers reviewed the data together in daily meetings in order to agree on areas to be further explored during upcoming interviews. This influenced the design of the research tool and allowed for purposive sampling of participants depending on the need. Assessment of saturation of data was also performed during the daily interviewers’ meetings. The data collection process was completed after 28 consecutive days.

The study tool was adapted from a qualitative interview guide used in a study done in Uganda by Agaro *et al*^18^ with modifications to suit the current context and designed to ensure that all aspects of the MDSR system were explored. Complementary methods (KIIs and FGDs) and purposive sampling of study participants allowed different aspects and different angles to be explored from the health providers in the facilities, health managers and government officials at the DEDs offices. Participants selected were diverse (table 1) and had different professions and different tasks within the MDSR implementation.

The interviews were conducted by three interviewers to reduce researcher-induced biases. All interviewers kept field notes that were used during data collection and analysis. The notes included the context description, dates, place and time of data collection, participants’ interaction during FGDs and response to understanding of questions. In each daily meeting the interviewers discussed issues that materialised during the interviews and went through their field notes. Some of the categories were identified and discussed during these meetings.

### Data analysis

All recorded interviews from KIIs and FGDs were transcribed verbatim for analysis. Thematic analysis as inspired by Braun and Clarke^19^ was used to develop themes that best described the findings in the data inductively. The first author read through the transcripts a number of times to understand the trend of the data. Themes that were identified during data collection (by AS and NS) in the field notes were also reviewed. Then open coding was done, followed by abstraction of codes to form initial subcategories. All the transcripts were in Swahili language, translation to English was done during coding where the Swahili speaking authors (AS) did the initial coding by reading through the Swahili transcripts and the codes generated were written in English. Microsoft Excel (2007) computer program was used during coding and formulation of categories and themes. The transcripts were written in one column of excel sheet then the code phrases were written in the next corresponding column to facilitate tracking of codes and original transcripts. The English generated codes were shared with other Swahili speaking authors (NS, AP and SM). Then the first (AS) and last author (MM) organised the initial subcategories and merged them to generate categories. The categories were then reviewed and collated to form subthemes, and these were in turn reviewed and abstracted into themes. Preliminary results were presented to the coauthors (NS, CH, SM and AP) for verification and consistency checking. Their comments inspired the final themes and interpretation.

### Patient and public involvement

This study was part of a lager project, which involved review of documents and interviews with health providers in Mtwara and Lindi regions. We did not involve patients as a study population. During the planning stage of the study, before ethical clearance was sought, the first author visited all districts in the two regions and held meetings with regional, district and facility health managers to get their opinions on how the study should be done, which documents are available and who and what issues needed to be explored. Their opinions were valuable during the planning and proposal preparation of the study. The results of the study will be shared with the health providers and managers in the two regions by oral presentations and a written report.

### Consent to participate

All participants received details about the study and its aims and were then asked to sign an informed consent form before the interview commenced. Confidentiality and secrecy were ensured by not using the names and positions of participants in the health system and conducting the interviews in rooms where no one else had access. The FGD participants were also assured that their findings would not be discussed with their managers in the facility. Use of identifying information was also avoided during report writing to ensure confidentiality. It was explained to participants that their participation or non-participation would not cause problems for themselves or their work. The interviews were audiorecorded with participants’ permission. The data (transcripts and audio recordings) were protected by saving them on the main researcher’s (AS) computer that only he had access to. The field notes were stored under lock and key and were only available to the researchers.

## Results

Two main themes emerged during analysis*: (*1*)* accomplishing by ambitions and (2) a flawed system. These were accompanied by seven subthemes: (under accomplishing by ambitions): desire to get it right, getting emotionally involved and producing change, (under a flawed system): Substandard implementation, hampered by organisational culture, assigning and avoiding blame and caught up in a demotivating environment (table 2).

**Table 2:** Themes, subthemes and categories emerging from the interviews

Categories	Subthemes	Themes
Active leadership by the book Rationalised review process Emphasising importance of time and context	Desire to get it right	Accomplishing by ambition
Thinking and acting for the baby Eye opener/exposure to new perspectives Feeling remorse and responsible	Getting emotionally involved	
Innovative solutions to lack of resources Discovering discrepancy of data Enhanced accountability Policy changes	Producing change	
Incomplete training cascade Poor dissemination and utilisation of the guideline Focusing on routine and formalities Using incomplete information	Substandard implementation	A flawed system
Selection process driven by hierarchy not need One man show Detached system Relying on development partners	Hampered by organisational culture	
Leaders expect themselves to be firm Leaders perceived as harsh and breech confidentiality Acting out of fear of blame Hot meeting/arguing and conflicts	Assigning and avoiding blame	
Need for continuous supervision Discouraged by lack of implementation of actions Repeated mistakes Discouraged by lack of incentives	Caught up in a demotivating environment	

## Accomplishing by ambition

This theme describes how the MDSR system was initiated and implemented with good intentions and with an ambition to make it work. This was accompanied by unexpectedly good outcomes that kept the provider motivated to implement the system.

### Desire to get it right

The health providers and managers emphasised the desire to make sure they get things right to implement the MDSR system in order to reduce maternal deaths. This was expressed by a perception that MDSR activities were facilitated by strong leadership and support from regional and district level managers. Furthermore, health providers and managers explained a rationalised review process by having multidisciplinary committees, using multiple sources of information, official invitation to committee membership and using recommended sitting arrangements in the meetings. There was also emphasis on timely notification and review, having a non-threatening environment in the meetings and an expressed intention to cooperate with other facilities.

…the support we get from above like from region, we get it, they do supervision according to their schedule… when they find gaps they tell us to improve… (FGD participant)

…when death occurs we have to cooperate, may be it has occurred in certain hospital, that woman might have came from another council, was sent as referral to this facility X… we plan a review meeting together with facility that referred to referral facility… even the dispensary where she attended ANC we also call them to take part in the meeting… (Key informant)

### Getting emotionally involved

Participants explained how initial MDSR discussions made health providers and managers act on emotional triggers to set up a regional-wide campaign to help newborn babies left in difficult conditions by the death of their mothers. It included donating baby formula and clothes from health providers and other stakeholders to help caretakers, who were mostly grandmothers.

…the mother is already dead, where will baby get mothers breast, the family is crying, the baby will continue to cry all its life. We strategized, who will wipe the tears of that baby. Therefore, we started campaign of “wipe my tears, help me and my mother live”… but through the program we have saved them, it is a good product of these meetings… (Key informant)

Even more important were the reported changes in attitude of health providers and managers in the way they approached maternal care. Recommendations from the system led to health providers, managers and government officials understanding how their own actions even outside labour/maternity ward (such as laboratory, pharmacy, theatre) can have a big impact on maternal deaths and the community in general. This prompted them to change the way they approached issues concerning maternal care in their work places.

…MDSR has helped us understand, at first it was a challenge as we took things simple. But when you participate you see the problems caused to a pregnant woman you wake up. It has changed us how we think of ward number 8 meaning maternity ward, all the (test) samples (from there) are considered as urgent. Therefore they have to be done as soon as possible… especially those with negative blood groups are scarce; we prepare to help them… (FGD participant)

### Producing change

Health providers and managers agreed that the implementation of the system led to innovative solutions for the notification process and lack of resources. These included extensive use of mobile phone technology, WhatsApp groups and short message service (SMS) in speeding up the process of notification of death. This notification system helped to discover discrepancies of data, such as differences in number of deaths between different levels of the health system. Lack of resources for different activities was solved by including funding for MDSR activities in annual plans and exchanging of supplies between facilities and districts.

…we have council coordinators, we have started WhatsApp group and we have normal messages group… we have put a strategy, when maternal death occurs, during day or night, even if 0200 hours at night, information must be sent immediately to regional level… there at regional… receive information on death even at 0200 hours… (Key informant)

…some district councils had financial problems and could not attend regional MDSR meeting… now every district council has to put aside a budget every year for their providers to attend regional MDSR meetings… (Key informant)

Participants revealed that the system helped to reinforce accountability of providers since health managers played a key role in making sure the system worked as intended by demanding feedback once a death was notified or reviewed. The health providers and managers also reported that the MDSR system facilitated policy changes at regional and zonal levels. Such recommendations were on contextual management of some conditions, staff management, referral system and issues of quality of care through training.

…this MDSR has helped us to get referral of patients, those that we thought they need further management. It has helped us to get referral to Muhimbili (National hospital)… We write referral. It has reduced number of deaths… we send them to Muhimbili… (FGD participant)

## A flawed system

This theme explains how health providers and managers thought the system was affected by detrimental organisational factors. They described contextual factors that provided multiple barriers for the intended implementation of the MDSR system.

### Substandard implementation

The cascade of training during initiation was perceived to have been incomplete. Health managers at regional and district health management teams were trained, but health providers reported to have never received formal training. Some health providers and managers reported reading the entire guideline, but it was generally poorly disseminated as other providers had never seen it and perceived it as difficult to access. Facility MDSR committees also explained they had little use of the guideline in the maternal death review meetings.

…I see a lot of shortcomings in MDSR issues because almost all members (MDSR committee members) have been included without any training… (FGD participant)

…therefore I got experience from meeting like that… I was never trained… I was not given the guideline… I have never seen the guideline to this day…(FGD participant)

The training and implementation of review meetings emphasised routines and formalities (such as filling forms) instead of critical reflections. The reporting form is recommended to be filled at the end of the meeting after all the discussions following the guideline. Heath providers explained how they focused a lot on the reporting form from the start to the end of the review meetings. Furthermore, the narrative summary, which is an important document used in the death review meetings, was affected by missing information in medical records and by the fact that it was written by a person unfamiliar with the case.

…we start (the review meeting) by following the flow of questions (in the maternal death reporting form) from top in heading of Ministry of Health, so we go item by item, step by step from number one to thirty something… (Key informant)

…The main problem when writing summary on the case is there are times you find the information are missing… some documents are lost for example the ANC card may be forgotten and taken home by relatives, some other information may be missing. It makes it difficult to prepare the summary… (FGD participant)

### Hampered by organisational culture

Health providers and managers expressed their dissatisfaction with the fact that decision making on MDSR issues were made by one person and selection of health providers for training was based more on hierarchy instead of what was needed on the ground.

The MDSR system was also described as a detached system, as most health providers and managers explained that it was not well integrated with other quality improvement teams and notification systems in the facilities. Another issue was lack of community–facility cooperation on issues of maternal death reviews. Facilities (especially hospitals) reported to have little or no cooperation with their surrounding community and depended on district leadership for this. The regional leadership played a key role in facilitating community visits to follow-up on maternal deaths.

…for us the MDSR is self reliant… when we decide issue of quality improvement we supervise and follow up ourselves… (Key informant)

…The cooperation (with community) I can say has many challenges… we are connected to community through the council…but there is poor communication… feedback to the facility or community is the main challenge… the hospital doesn’t have (connection to community), we rely on the council managers… (Key informant)

The initial training and regional quarterly review meetings relied to some extent on financial and technical support from a developmental partner who was working on maternal and child health in the regions. This hindered the process of cascading the training down and organising the regional meetings when the developmental partner left at the end of the programme. The regional management came up with innovative idea to address some of these challenges.

…the challenge in the beginning was funds; we needed funds to conduct regional meetings… some did not attend due to lack of funds… that’s why development partner came and help, those from GIZ (Deutsche Gesellschaft für Internationale Zusammenarbeit)… they were able to pay from council and regional levels… they were paying invited members… but later we were able to stand on ourselves… (Key informant)

### Assigning and avoiding blame

Health managers at regional, district and facility levels expected themselves to be firm, especially in face of negligence and disregard of procedures. This caused breaching confidentiality of meetings and the use of MDSR information for punishing health providers. Fear of blame in the meetings caused health providers to avoid attending meetings and they testified to sensing tension when invited to one. In some cases it resulted in falsification of documents, late notifications and concealing cases of death.

…there is time we say don’t point fingers, but there are times you can say there is negligence that is too much… if you are not a bit harsh nothing goes well. Therefore not pointing finger, staying silent for everything, this for some issues that are very sensitive, and you can see it clear negligence I don’t agree… (Key informant)

…if it (death) happen because of negligence the provider is called and is told his/her shortcomings even though it was discussed there (in the meeting)… he/she will be called by leadership and is told… (FGD participant)

…then it has breach guidelines, because we have started using information in another way. If we have decided to discuss as XXX, it means we are hiding those names, we should continue with XXX… (FGD participant)

…at the end of it, later this person will come and hide information because a death will happen and he/she will tear papers (in patients files) where she worked and write it again (with changed information)… (FGD participant)

### Caught up in demotivating environment

Health managers expressed their frustration with the poor attitude of some providers in implementing MDSR activities and the action plans created during the reviews. They perceived that this necessitated continuous or frequent supervision. Action plans were also affected by lack of financial resources for implementation, long procurement procedures and bureaucratic processes in asking for funds from higher authorities. Lack of commitment to implement these actions also meant the same mistakes were repeated, which caused additional deaths. Health providers also expressed being discouraged by the lack of incentives to participate in MDSR meetings, which took a long time, sometimes starting after work hours, on weekends or on their off-duty days.

…there are some people with a lot of challenges, you will yourself talking a lot but tomorrow repeats (same problem) every time. You talk and talk but it’s a problem… you are forced to ask in-charge to follow everything he/she does, everything done… (Key informant)

…the issue of recognition to members should be mentioned, not just wait for death… it is connected directly with issues of lack of motivation, some other day they should provide certificate for recognition, it will energize us… (In the MDSR meeting) if there is little water or something (money) none of the members will leave… (FGD participant)

## Discussions

Our findings suggest a mismatch between the ambition, execution and outcome of implementing the MDSR system in Tanzania. The main aim of introducing MDSR was to improve the quality of care. This was to be done by learning from the causes of maternal deaths. Its implementation builds on the involvement of multiple stakeholders at different levels of government and the health system to fulfil their responsibilities. This means implementers should have the right desire and motivation in order for the system to work. Literature also suggests that strong leadership, committed health providers, government support and the coordinated approach of different stakeholders in the health system are important in implementation of MDSR systems.^11 20 21^ Health providers and managers at different levels of the MDSR system in our study expressed a desire to make sure the system works as intended. The MDSR system implementation, however, faced challenges such as lack of training for most providers, poor utilisation of guidelines, lack of reflection during the review process, missing information in medical records and poor integration with other systems. Furthermore, poor implementation of action plans and lack of incentives discouraged some providers from taking part in MDSR activities. These challenges have also been echoed in other studies done in low-income and middle-income countries.^11 15 22–24^ Literature shows that improving knowledge and skills of providers and integrating MDSR with other systems and stakeholders will improve the efficiency of the system in identification, notification and review of deaths.^14 25^

The success of MDSR depends on honest and open discussions about the events that preceding each death. This means a non-threatening environment for providers to feel safe to discuss the events must be created. A non-threatening atmosphere can only be ensured by making sure MDSR information is kept confidential and that no names connected to the specific case are used during the meetings.^6^ Studies in Malawi and Ethiopia have all reported how the issue of fear of blame and avoiding personal accountability has affected the implementation of MDSR.^13 21 26 27^ The culture of blame could lead to falsification and missing records as explained in this and other studies. Health providers and managers need appropriate training that specifically addresses the culture of blame. Health managers should understand that the culture of assigning blame affects the system negatively, even though their managerial positions incline them to appear strict to providers.

The attribution theory also explains that peoples perceptions about the root of the problem influences their response to these events.^28 29^ Our study explains how health managers put more emphasis on internal characteristics or factors of health providers such as attitude towards work when attributing causation instead of also taking external factors into consideration. The MDSR system is somewhat related to Weiners explanations that attribution of causality is done not only for understanding purposes but also in order to control future events.^28^ Weiner implies that health providers are more likely to change behaviour if they attribute the cause to their lack of skills when they have all the necessary resources.

The health managers should create a supportive environment for providers to take part in MDSR activities without fear of blame, and apply a systems-thinking perspective when investigating what has gone wrong. This approach entails moving from individual models, to looking at how different characteristics within systems are connected to each other and the relationships between systems.^30 31^ In this concept, changes in one element can have a ripple effect across others that can in turn lead to positive or negative feedback across the whole system. For example, addressing the issues of blame will improve notification, documentation and the quality of the review process. This can be accomplished by creating a system where disciplinary accountability mechanisms for negligence are kept separate from MDSR activities.

Even though its implementation was clouded by many challenges, the MDSR system was perceived to have exhibited sporadic impact on important issues such as changes in policy, increased accountability, improved service provision and personal provider behaviour, as well as innovative solutions to overcome resource limitations. This implies that the commitment shown by managers and providers in implementing the system were not in vain. Seeing actual improvements acted as one of the motivating factors to sustain the system, even though it still faces many challenges. In Ethiopia and Nigeria, the MDSR system was reported to have great impact on issues relating to quality improvement. Training of staff, provision of guidelines and job aids, establishment of operating theatres and intensive care units, sensitising staff to prevent deaths and creating blood transfusion mechanisms were all reported to be successes stemming from the implementation of MDSR recommendations in these settings.^10 32^ Addressing challenges facing the system would have far reaching effects on the efforts to reduce maternal mortality. This will in turn make MDSR more effective in improving quality of care and reduce maternal deaths.

### Implications for the system

The MDSR system faces challenges in implementing all of its steps within the cycle. The identification and notification process was explained to be done on time through WhatsApp and SMS groups. Still, the notification of deaths was hindered by lack of commitment, fear of blame, lack of integration, and missing the deaths from other wards and the community. Said *et al* revealed that maternal deaths notified through MDSR in the study area were fewer compared with estimations by other national and international systems.^14^ The identification system should be more comprehensive to include all facility and community deaths. We suggest including community health workers in the identifying and notifying of community deaths. Each hospital should have one focal person to identify all suspected maternal deaths in all wards.

There was a rationalised review process, where deaths were reviewed on time, in collaboration with facilities, using multiple sources of data and in multidisciplinary committees. The process was highly affected by badly written narrative summaries, breach of confidentiality, non-utilisation of the guidelines and blame culture. This also explained the findings in the study by Said *et al* which reported poor identification of three delays by the committees. It also confirms the findings from another study which showed that written summaries used in MDSR were not comprehensive.^15^

The most important step of the MDSR system is the implementation of action plans. Even though the system did not have a systematic way of tracking implementation of each action plan, health providers and managers reported that most were implemented. They also explained evidence on the impact of implementing these actions at facility, district, regional and zonal levels. Since there was no tracking system it is difficult to judge the extent of how these actions were implemented and the true impact of it.

### Limitations of the study

The main limitation of this study was that we did not include implementers at national level and higher-level facilities like zonal and national hospitals. This is due to the nature of the study that sought to explore implementers’ perspectives at regional, district and facility levels. The inductive approach used in this study could be a limitation and also strength. The strength of this method is that it has been described to be best for describing observations and experiences like in our study.^33^ The results from inductive approach also are inferred from the data and not limited to specific theory. On the other hand inductive studies are limited by the fact there is uncertainty on the repeat of occurrence of the findings and reaching of saturation.^34^ The findings from our study could have been different if the analysis was done by different researchers. It is also argued that in inductive approach there is always an element of deductive when formulating categories and themes.

The results of this study may also have been affected by the first author’s preunderstanding of the MDSR system, both from his experiences as a clinician, his training with MDSR and from previous studies conducted. The author may have used findings from other studies and work he has been involved with to interpret some of the data in this study. In order to reduce this bias, other authors and data collectors were involved in the study design, data collection and analysis to make sure the interpretations were derived from the data. Attempts were made during data analysis to delineate the authenticity of abstractions made.

The fact that the FGD participants were selected by involving the health managers could also have affected the way health providers described their experiences and perceptions. This could be due to fear of blame from managers and providers self interests in concealing their own shortcomings in implementation of the system. We sought to minimise this by working closely with managers during selection of providers and made sure the selected participants met our inclusion criteria by directly enquiring from them. Before commencement of FGDs a demographic checklist was also used to make sure participants met the criteria and none of them were managers in their work place. We further ensured audio visual secrecy of the discussions and made sure the health managers were not in or around the venue of the discussions. We also explained to the participants that the findings of their discussions will not be discussed with their managers and confidentiality will be ensured in writing the report and this manuscript.

### Trustworthiness

The trustworthy criteria were inspired by Lincoln and Guba and were used during the planning, data collection and analysis phase. To ensure credibility of the results efforts were made to ensure all interviewers had sufficient knowledge on the MDSR system and what was required for the study. Several meetings were held between interviewers during the planning stage, data collection and analysis. Meetings were also held between researchers and interviewers to discuss the protocol, research tool, data collection and analysis plan. To address reproducibility of the findings, we attempted to have clear explanations of all research methods and protocols. This was discussed and reviewed several times by all researcher and interviewers. Confirmability was provided by triangulating the data collection methods (KIIs and FGDs), study participants and interviewers. Reflexivity was also explained in the limitation section above. The findings can be applied in other settings as participants were purposively selected and snowballing was employed to add more participants as required. Data saturation was also checked during daily meetings and the analysis phase.

## Conclusions

The implementation of the MDSR system in Tanzania faces systemic, contextual and internal challenges. However, our results also indicate that there is a willingness and commitment to improve service delivery and avoid maternal deaths among healthcare providers. This asymmetry between the expressed intentions and ambitions of the MDSR system and the accounts of the short-comings of the actual implementation identifies possibilities for improvement. Policy planners and decision-makers should capitalise on the willingness to do well that is evident from the interviews and address the contextual barriers that hamper the MDSR system. Health providers in facilities should have proper MDSR training to better understand their roles in the system. Implementers should be enabled to access and encouraged to use the MDSR guidelines. The issues of blame culture should be addressed by managers, they should be held true to their promise of upholding confidentiality and remember to appreciate providers’ efforts to reduce maternal deaths.

## Data Availability

The data (audio recordings and transcripts) are available from the correspondence author on request.
